# Deep Learning Model to Predict Ice Crystal Growth

**DOI:** 10.1002/advs.202207731

**Published:** 2023-05-17

**Authors:** Bor‐Yann Tseng, Chen‐Wei Conan Guo, Yu‐Chen Chien, Jyn‐Ping Wang, Chi‐Hua Yu

**Affiliations:** ^1^ Department of Engineering Science National Cheng Kung University No. 1, University Rd. Tainan 701 Taiwan

**Keywords:** casting, dendritic structure, generative model material, reinforcement learning, solidification

## Abstract

The demand for highly specific and complex materials has made the development of controllable manufacturing processes crucial. Among the numerous manufacturing methods, casting is important because it is economical and highly flexible regarding the geometry of manufactured parts. Since solidification is an important stage in the casting process that influences the properties of the final product, the development of a controllable solidification process using modeling methods is necessary to create superior structural properties. However, traditional modeling methods are computationally expensive and require sophisticated mathematical schemes. Therefore, a deep learning model is proposed to predict the morphology of the dendritic crystal growth solidification process, along with a reinforcement learning model to control the solidification process. By training the deep learning model with data generated using the phase field method, the solidification process can be successfully predicted. The crystal growth structures are designed to be altered by adjusting the degree of supercooling in the deep learning model while implementing reinforcement learning to control the dendritic arteries. This research opens new avenues for applying artificial intelligence to the optimization of casting processes, with the potential to utilize it in the processing of advanced materials and to improve the target properties of material design.

## Graphical abstract

This work investigates a design framework that integrates machine learning to control and optimizes dendrite structures. A ResNet‐U‐Net surrogate model is successfully developed and trained to predict the phase evolution of dendrite structures. Meanwhile, the deep deterministic policy gradient is implemented to manage simulation parameters, ultimately generating structures with desired properties.

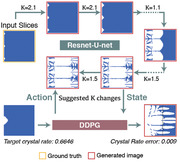

## 1 Introduction

Casting and solidification are often used to fabricate complex shapes in the commercial processing of many materials because of their flexibility and practicality. However, the controlling mechanisms for casting processes have not yet been fully elucidated, making process control dependent on empirical formulae. Consequently, a variety of numerical methods have been used to establish laws and simulate the casting process, for which an understanding of the science of solidification is of paramount importance to interpret the macro‐and microscopic changes that occur. In addition to its applications in the traditional foundry industry, solidification is also the basis for many new processes and materials such as semi‐solid casting,^[^ ^1^ ^]^ laser melting,^[^ ^2^ ^]^ powder atomization,^[^ ^3^ ^]^ metal matrix composites,^[^ ^4^ ^]^ and bulk metallic glass.^[^ ^5^ ^]^

Since dendritic structures are commonly observed in metal solidification,^[^ ^6^ ^]^ simulations often focus on their temporal‐spatial development. The growth of dendritic morphology affects the shape of the pores in a porous material and changes its properties. There are several methods used in solidification simulations, such as phase field, cellular automata,^[^ ^7^ ^]^ and molecular dynamics,^[^ ^8^ ^]^ of which phase field is the most appropriate method for the simulation of dendritic morphology. Derived from thermodynamics, phase field models represent the solid–liquid interface as a continuous transition layer with a finite thickness between the two phases using additional variables, which avoids the need to track the phase boundaries. Although this method can simulate dendritic growth with high precision, it requires sophisticated mathematical schemes, including partial differential equations and numerical analysis, making the simulation computationally expensive.

In recent years, machine learning has been used in numerous data processing tasks. The wide range of algorithms and modeling tools associated with it has been successfully introduced into materials science^[^ ^9^ ^]^ and physics.^[^ ^10^ ^]^ Scientists can exploit the power of artificial intelligence (AI) to analyze data and extract information to make predictions or decisions. AI algorithms can process images of materials as input and predict their complex stress or strain fields,^[^ ^11^, ^12^ ^]^ as well as predict the dynamics of crack propagation.^[^ ^13^, ^14^ ^]^ AI also has potential applications in various physics‐informed problems due to its inherent ability to discover hidden correlations between data and solve high‐dimensional problems.^[^ ^15^ ^]^ Reinforcement learning (RL), an AI subfield that uses agents to perform optimized actions in specific environments to maximize designed rewards, has emerged as a candidate for the optimization of material design schemes. By transforming the design space of materials into a controllable environment, RL can use the interaction between agents and the environment to optimize the design of superior materials.^[^ ^16^ ^]^

Numerous successful precedents demonstrated the benefits of integrating AI with phase field simulations. These included the utilization of machine learning to construct analytical expressions,^[^ ^17^ ^]^ the representation of high‐dimensional free energy surfaces using deep neural networks,^[^ ^18^ ^]^ the application of principal component analysis for the analysis of the outcome of phase field modeling,^[^ ^19^ ^]^ the prediction of the time evolution of phase field modeling through neural networks,^[^ ^20^, ^21^, ^22^ ^]^ the integration of neural networks into the design pipeline of electromigration parameters,^[^ ^23^ ^]^ etc. These excellent results show the significant potential of employing AI algorithms to advance the study of phase field models. Despite previous successful works in predicting the time evolution of phase field modeling, our framework extends this structural prediction and included controllability to the neural network to adjust the outcome. By utilizing the U‐net architecture, which is conventionally implemented for segmentation problems, we achieved the spatial and temporal predictions, while being able to control the output conditions using additional channels in the input image. Our framework further incorporates RL to achieve the design of phase field models with target properties.

**Figure 1:**
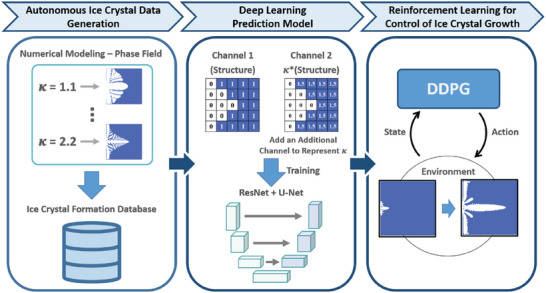
The framework of this study. The phase field method was applied to generate the dataset with different 𝜅 conditions to obtain diverse ice formations. The growth process and the ice morphology varied at each timestep due to the different initial temperatures and conditions. Subsequently, the U‐net‐based model was trained and implemented as the deep learning model for prediction. Finally, the pretrained model was used as the environment for the RL agent to train and realize control over the ice crystal rate.

Our aim is to use deep learning to acquire knowledge of the underlying relationship between the development of dendritic solidification morphology and control morphology by adjusting the input parameters of a deep learning model using RL. Our research framework is shown in **Figure** **1**. The two essential components of the framework are the deep learning model for the prediction of ice crystal growth and the RL agent that controls ice crystal growth. Our deep learning prediction model, which combined the network architecture of both ResNet and U‐net, is trained on a dataset that consists of a set of images at different time steps of simulated dendritic growth with spatial associations. In combination with additional condition parameters, the deep learning model can generate different ice crystal growth outcomes under different conditions, thereby developing correlations between the control parameter and the predicted output. By using the prediction model of the crystal growth process as the RL environment, the RL agent can learn to optimize the condition parameters to achieve the designated goals, which will ultimately facilitate the intentional design of ice crystal growth.

## 2 Experimental Section

### 2.1 Phase Field Model for Ice Solidification

The phase field method, which is based on the Ginzburg‐Landau theory^[^ ^24^ ^]^ and uses differential equations to model the effects of diffusion, ordering potential, and thermodynamic drive with specific physical mechanisms, was used to generate the necessary dataset for the deep learning prediction model. With this method, the instantaneous temporal and spatial states of the system under consideration can be obtained, and the spatial arrangement of phases and defects enables the description of a microstructure with a different composition and structural features. The interaction of interfaces in the complex processes that occur during phase transformations require nonlinear equations to represent them. The phase field method overcomes the difficulty in solving these nonlinear equations by adding different interface descriptions into its formulation.

The phase field model used in this study is based on the dendritic solidification model proposed by Kobayashi.^[^ ^25^ ^]^ We chose this model for its efficiency and simplicity, which facilitated the generation of training data with ease. Our primary objective is to develop an appropriate deep learning model that serves as a surrogate to expedite the inference time of the phase field model simulation. Therefore, selecting a phase field model with a faster simulation time is crucial for making adjustments to our deep learning model effectively. This simple phase field model incorporates the interfacial anisotropy that occurs in the solidification of a one‐component system. The Kobayashi model explains the qualitative relationships between crystal geometry and the physical parameters accompanied by noise, which is a significant influencing factor in the detailed development of the side branch structure of dendrites. The model incorporated two variables: One of the variables is the parameter of the nonconserved phase‐field parameter, *φ*(*r*, *t*), which has a value of one for solid and zero for liquid. The other variable, the temperature field *T*(*r*, *t*), is one of the controlling factors of the phase field model and it evolves as the solidification progresses. The boundary condition in our work is set as the zero flux boundary condition.^[^ ^25^ ^]^ For the parameters mentioned above, *r* represents the spatial position and *t* is the time. The evolution of the temperature field *T*(*r*, *t*), is derived from the conservation law of enthalpy as

(1) $$\frac{\partial_{T}}{\partial_{t}}=\nabla^{2}\;T+\kappa \frac{\partial_{\varphi }}{\partial_{t}}$$

(2) $$\kappa \;=\frac{\Delta H}{C_{p}\;\cdot \;\left(T_{m}-T_{c}\right)}$$

where *T* is the nondimensionalized temperature for which the characteristic cooling temperature is zero and the equilibrium temperature is one, 𝜅 is the dimensionless latent heat and is directly proportional to the latent heat and inversely proportional to the strength of the cooling, *C_p_* is the specific heat at constant pressure, Δ*H* is the latent heat, *T*_m_ is the melting temperature, and *T*_c_ is the crystallization temperature. The derivation of 𝜅 is based on the literature^[^ ^26^ ^]^ and the quantity is considered to represent the degree of supercooling. 𝜅 is positively correlated with the crystallization temperature and melting temperature, which is based on an actual water‐ice crystallization experiment.^[^ ^27^ ^]^ The higher the value of kappa, the more heat is released from the diffusion layer at the interface.^[^ ^28^, ^29^, ^30^ ^]^ For simplicity, the diffusion constants are set to be identical in both the solid and liquid phases. In addition, Kobayashi's model does not rigorously define the sharp interface limit as *ε* → 0, but the results of Kobayashi's model show that it converges to the expected classical sharp interface result. In our simulation, *ε* is close to 0.01 in the 10 252 time steps.^[^ ^31^ ^]^ All the phase field simulations in this study were performed using MATLAB, and the total number of timesteps in the simulation was set as 44. The parameters used in phase field simulations are shown in **Table** **1**.^[^ ^25^ ^]^

**Table 1:** The parameters used in phase field simulations

Parameters	Value
Domain size	300 × 300
Grid spacing	0.03 mm
Number of time steps	10 252
Time spacing	0.0001 s
Releasing time (*τ*)	0.0003 s
Mean anisotropic gradient energy coefficient ($\bar{\varepsilon }$)	0.01
Anisotropic strength (*δ*)	0.02
Initial angle (*θ*_0_)	0.0
Equilibrium temperature (*T*_e_)	1.0 K
Dimensionless latent heat (𝜅)	1.1–2.1

### 2.2 Autonomous Ice Crystal Data Generation

The dataset used in this study was generated autonomously using phase field modeling from a simulation of the development of ice morphology. The autonomous generation included three different control variables: 1) the variation of 𝜅 during the simulation process, 2) the number of changes in 𝜅 during the simulation process, and 3) the number of ice nuclei. Using the in‐house designed data generator, different combinations of the control variables could be used as parameters for the generator, and hence, the phase field method operates under the variously derived conditions, creating image sets for different cases of ice morphology development.

Firstly, the variation of 𝜅 ranged from 1.1 to 2.2. Within this range of 𝜅, the ice crystal formation showed a variety of different outcomes. The ice crystal has a “plate” structure when 𝜅 is 1.1. As 𝜅 increases, this structure will gradually become dendritic and, finally, it will become a “needle” at 2.1. Branches grow with different tendencies according to 𝜅 until they hit boundaries. When branches contact these boundaries, they interact and cause ice crystals to grow along the wall. The ice crystal has a “plate” structure when 𝜅 is 1.1. As 𝜅 increases, this structure will gradually become dendritic and, finally, it will become a “needle” at 2.1. By varying the value of 𝜅 between 1.1 and 2.1, we can obtain ice crystals of different shapes. For the model to learn to grow into ice crystals of different shapes and use them for subsequent design, we generated data with 𝜅 values all within the range of 1.1 to 2.1, which limits the possible combinations and allows the deep learning model to have a higher convergence rate. Secondly, because 𝜅 is strongly related to the results of the simulation and adjusted to real‐world experiments to control the shape and porosity of ice, changing the value of 𝜅 at different timesteps during the simulation can result in different outcomes. Hence, we designed three change patterns for 𝜅 during the simulation process, where 𝜅 is to be varied at evenly distributed timesteps. Since there was a total number of 44 timesteps, the three change patterns used can be described as 1) constant 𝜅 throughout the simulation, 2) 𝜅 changes once during the simulation at timestep 18, and 3) 𝜅 changes twice during the simulation at timesteps 12 and 24. Lastly, the total number of ice nuclei used ranged from one nucleus to three nuclei. The nuclei were placed evenly along the left‐hand side of the initial simulation frame, which allowed the nuclei to grow toward the righthand side. In all these cases, each complete cycle of the ice crystal growth process contained 44 pictures, and each picture contained 128 × 128 pixels. To allow the deep learning prediction model to learn the relationship between the intrinsic parameter 𝜅 and the development of the ice crystal, an additional channel was concatenated to represent the set 𝜅 value, for which its frame was multiplied by 𝜅, i.e., this additional channel had a data representation similar to that of the original simulation frame, but the pixel values were increased by a factor of 𝜅. The ice crystal formation database was thus established following the process.

### 2.3 Statistical Analysis

Phase field model simulations were carried out in MATLAB, with 10 252 time steps (i.e., 10 252 frames), equivalent to 1.0252 s, used to generate the evolving structures for each simulation. The simulation domain for forming the dendrite structures consists of a 300 × 300 square cell grid, each cell measuring 0.03 mm in length, resulting in a total domain size of 9 mm^2^. To reduce the memory requirements for training and improve the distinction between each frame, these image frames were evenly divided into 44 frames, promoting better ResNet‐U‐net convergence. The total dataset consisted of 2073 simulation trials, with each trial containing a batch of 44 frames. Within the total dataset, 80% (1658 batches) were used for training the ResNet‐U‐net and 20% (415 batches) were used for validation.

For training and benchmarking our deep learning model, the time evolution of modeling results and simulation conditions (i.e., 𝜅) were extracted as single‐channel binary images and text‐based information. The extracted data was then imported into custom Python scripts for preprocessing and training. Our ResNet‐U‐net input images comprised two separate channels. The first channel represented the 2D dendrite structure, saved as a binary image with the structure represented by 0 s and the background by 1 s. To incorporate 𝜅 into the deep learning model, an additional channel was appended to the image channel to represent the simulation conditions. This channel was formed by directly multiplying 𝜅 with the first channel, resulting in an image similar to the first channel, with the background representing the 𝜅 value for each simulation frame. This additional channel was included as it can improve the accuracy of the ResNet‐U‐net and demonstrates the feasibility of using additional channels to control U‐net outcomes.

## 3 Results

### 3.1 U‐Net Enabled Ice Crystal Prediction

**Figure 2:**
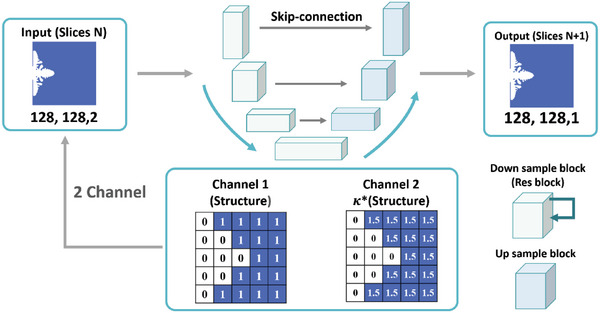
ResNet‐U‐net architecture of the prediction model. The input data contains two channels, one for the original simulation frame and the other for the 𝜅 representation of the original simulation frame, resulting in an input dimension of (128, 128, 2). The input data is then passed through the ResNet‐U‐net model to predict the ice crystal formation in the next simulation frame, resulting in an output dimension of (128, 128, 1).

We proposed a deep learning model capable of predicting the evolution of the ice morphology using a phase field model. To mimic the characteristic of the phase field method, the designed model should have spatial prediction abilities as the phase field method outputs ice morphology using spatial representations. To meet these requirements, we used a combination of two types of machine learning models, the residual neural network (ResNet) and the U‐net, as shown in **Figure** **2**.

The U‐net architecture can be divided into two paths. The first is the contracting path, which performs multiple convolutions and pooling of an image to reduce its size. This will also reduce the number of calculations required and enhance the specific features and noise reduction effects. The second is the expansion path, which restores the original scale through deconvolution. During the up‐sampling process, the original feature maps of the same layer are connected to ensure that any information lost during down‐sampling is preserved as much as possible. Retrieving lost features by splicing is the most important aspect of U‐net.

ResNet is used for problems where the accuracy saturates and drops rapidly as the depth increases. When introduced through a new structure, it allows the network to have more depth. For a stacked‐layer structure, the original learned feature is recorded as *H*(*x*) when the input is *x*, and the expectation is that it can learn the difference between the input and target features, where the difference is the residual. The corresponding relationship is

(3) $$F\;\left(x\right)=\;H\left(x\right)-x$$

The original learning feature *H*(*x*) can be regarded as *F*(*x*) + *x*, and residual learning is easier to implement than using the original feature directly. This also avoids degrading the network performance when the residual is zero, preventing degradation with increasing depth.

We expected to be able to predict ice crystal formation by giving the initial nucleation locations and numbers. In addition, we also expected to be able to control the shape of ice crystals by specifying conditions for ice crystal design during the growth process. U‐Net is often applied to solve image segmentation problems and the process of ice crystal growth can be viewed as a problem like that of edge detection. Each growth (increase in ice crystals) is based on the original image. Therefore, in addition to the ice crystal structure, we included 𝜅 (see the Experimental Section for details), as a conditional input to the model to control the growth.

To train the model to predict ice solidification under various 𝜅 conditions, adjustments were made to the training data. During the data generation process, an additional channel was concatenated to the original channel to depict the ice crystal formation. This channel was added to the training data to allow future predictions to gain control of 𝜅 when generating ice crystal formations, since the differences in 𝜅 could generate very different results. At each timestep of the phase field method, we converted the output image into a binary representation. Then, by multiplying the image using the simulated 𝜅 value, the new channel could be acquired and concatenated onto the original channel. The training process involved separating the data into pairs, where one of the datapoints was randomly selected from the dataset and the other was the next frame of the previously mentioned datapoint. By using the successive frame as the ground truth output of the U‐net, the presented U‐net was able to learn the relation between frames and could successfully generate the corresponding frame.

### 3.2 Ice Crystal Growth Prediction

To test the predictive ability of the proposed model, we first trained the model using various 𝜅 parameters. That is, the model contained additional parameters to control 𝜅 during the simulation process, similar to that of conditional generative adversarial networks. This model served as a generative model for controlling the generated images conditionally. The model we used in this study used the structure of the ice crystal and the 𝜅 value as input to predict the shape of the ice crystal in the next frame. We used a combination of binary cross entropy (BCE) and dice loss as the loss function to judge the predictive performance of the model. The loss in the training and validation datasets is approximately 0.01, and the accuracy for the training and validation data sets is above 99%. We also used the Dice coefficient to judge the correctness of the model's image predictions. The Dice coefficients of the training data set and the validation data set are approximately 0.99, which indicates that the model has high predictive ability.

**Figure 3:**
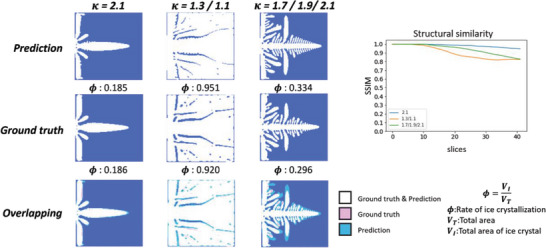
Predictions for the formation of ice crystals with a single nucleus. The prediction results where the top left rows show the predictions, the middle rows show the ground truth acquired using the phase field method, and the bottom rows show the overlapping visualizations of the prediction and ground truth. On the right is the structural similarity of the iterative process.

To generate different ice crystallization rates, different cases of 𝜅 variation and the number of crystal nuclei were applied to the dataset. Here, we show examples with one to three nuclei and one to three segments having a different number of 𝜅 changes. The results for the single crystal nucleus prediction are shown in **Figure** **3**, where Phi is the rate of ice crystallization. In the overlapping images, white indicates a correct prediction (i.e., prediction and ground truth overlap), pink indicates only the ground truth, and blue indicates only the prediction. The error in the results ratio for the same 𝜅 is 0.001. When there are two changes, 𝜅 varied from 1.3 to 1.1, with a good degree of accuracy and error ratio of 0.031. When there were three changes, 𝜅 changed from 1.7 to 1.8 to 1.9. Although the predicted results are redundant, the final error is 0.038 and the trend is the same. The structural similarity index measure (SSIM) is an index that represents the similarity between two images. Using SSIM, it can be observed that initially, the similarity between each case is highly similar. As the time step progresses, the SSIM scores vary, and are consistently high in accuracy when 𝜅 remains unchanged.

**Figure 4:**
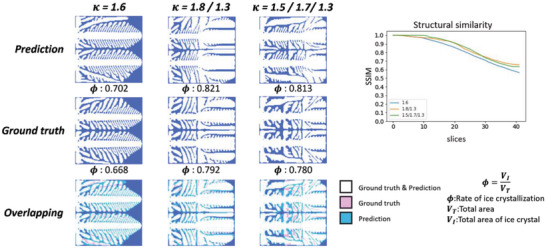
Predictions for the formation of ice crystals with double nuclei. The prediction results where the left top rows show the predictions, the middle rows show the ground truth acquired using the phase field method, and the bottom rows show the overlapping visualizations of the prediction and ground truth. On the right is the structural similarity of the iterative process.

The results of the prediction of the double‐crystal nucleus are shown in **Figure** **4**. The ratio error in the results for the same 𝜅 is 0.032. In the case of two‐stage change, 𝜅 varied from 1.8 to 1.3. The structure changed from dendritic to plate‐shaped, and the ratio error is 0.029. The three‐stage change has 𝜅 varying from 1.5 to 1.7 to 1.3 and it has the same overall trend with an error of 0.033, except for the middle stage, which has a larger error. Although the accuracy of structural similarity is lower than that of a single nucleus, the overall trend of white can be seen in the overlapping figure.

**Figure 5:**
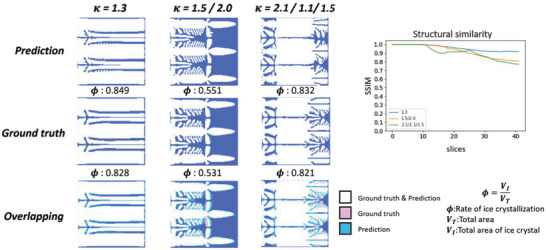
Predictions for the formation of triple nuclei ice crystals. The prediction results where the left top rows show the predictions, the middle rows show the ground truth acquired using the phase field method, and the bottom rows show the overlapping visualizations of the prediction and ground truth. On the right is the structural similarity of the iterative process.

In the case of a three‐crystal nucleus shown in **Figure** **5**, the resulting ratio error for the same 𝜅 is 0.032. In the case of two‐stage change, 𝜅 varied from 1.5 to 2.0. The structure changed from dendritic to needle‐shaped, and the ratio error is 0.02. The three‐stage change has 𝜅 varying from 1.5 to 1.1 to 2.1, and the second and third 𝜅 changes are significantly different from those of 1.1 to 2.1. The model can also predict the same trend with an error of 0.011. In the case of triple nuclei, the structure is similar and the accuracy is highest when 𝜅 remains unchanged. The cases with 𝜅 variation also have an accuracy of around 0.8.

### 3.3 Porosity Control Using Deep Deterministic Policy Gradient

**Figure 6:**
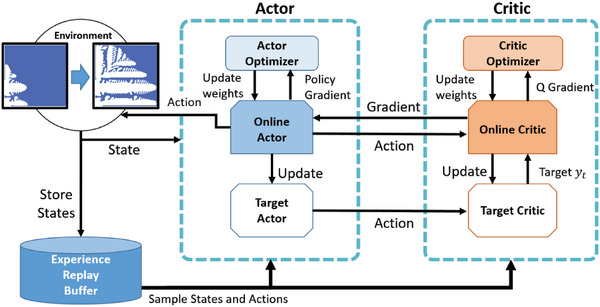
DDPG framework. By taking actions in the environment while storing states in the experience replay memory, the actor and critic networks of the DDPG can be trained by sampling the states and actions in the replay buffer. The stability of DDPG can be improved using the target networks.

We propose an RL approach to achieve porosity control. The deep deterministic policy gradient (DDPG)^[^ ^32^ ^]^ is an actor‐critic RL algorithm that is model‐free and based on the policy gradient. At each timestep *t* of the DDPG, the actor network receives the state *s_t_* of the environment and outputs a corresponding action *a_t_* through the trained policy function. Then, the critic network will evaluate the quality of *a_t_* at the given *s_t_*, and the state of the next timestep *s*_*t* + 1_ can be acquired by performing *a_t_* on *s_t_* in the environment *E*. This algorithm contains two sets of actor and critic networks, the online actor network *μ*(*s*|*θ* ^*μ*^) with the online critic network *Q*(*s*, *a*|*θ* ^*Q*^), and the target actor network *μ*′(*s*|*θ* ^*μ*′^) with the target critic network *Q*′(*s*, *a*|*θ* ^*Q*′^) where *θ* represents the network parameters. The target networks are time‐delayed versions of the online networks, which slowly track the weights and parameters of the online networks using soft updates rather than directly copying them, as shown below

(4) $$\left\{\begin{array}{c} \theta^{Q^{\prime }}\leftarrow \tau \theta^{Q}+\left(1-\tau \right)\theta^{Q^{\prime }}\\ \theta^{\mu^{\prime }}\leftarrow \tau \theta^{\mu }+\left(1-\tau \right)\theta^{\mu^{\prime }} \end{array}\right.$$

where *τ* is the soft‐update factor. With the target network, the *Q* value can be calculated using the Bellman equation, as follows

(5) $$y_{t}=r_{t}\;+\gamma Q^{\prime }\left(s_{t+1},\mu^{\prime }\left(s_{t+1}|\theta^{\mu^{\prime }}\right)|\theta^{Q^{\prime }}\right)$$

where *r_t_* is the reward function at timestep t and *γ* is the discount factor. This mechanism allows the evaluation of the *Q* value to be less dependent on online networks, which can effectively improve the stability of the trained actor and critic. Using the *Q* value, the critic network can be trained by minimizing the squared difference between *Q*(*s_t_*,*a_t_* |*θ* ^*Q*^) and *y_t_*, and the target network can be trained by minimizing *Q*(*s_t_*,*a_t_* |*θ* ^*Q*^). Furthermore, a replay buffer mechanism is used to allow the algorithm to benefit from the learning in previous training episodes. At each roll‐out, the episode tuple (*s_t_*,*a_t_*,*r_t_*,*s*_*t* + 1_) is saved in the replay buffer. When the networks are to be trained, mini‐batches of episode tuples can be sampled randomly from the replay buffer and used to train the networks. Furthermore, a normally distributed noise term is directly added to the action to encourage exploration. The DDPG framework is shown in **Figure** **6**.

To achieve porosity control using DDPG, we use the prediction deep learning model as the RL environment and formulate the problem in the form of state, action, and reward. Because the objective is to control the porosity of ice crystal growth, we set the target of DDPG as the rates of ice crystallization, which is complementary to the porosity and can be calculated using

(6) $$\phi \;=\frac{V_{I}}{V_{T}}$$

where *V*_I_ is the area of ice crystals in the simulation frame and *V*_T_ is the total area of the simulation frame. With the target of the problem set, we designed the state to be related to the target defined by two parameters: 1) the difference in the ice crystallization rate between the current simulation frame and the target ice crystallization rate, Δ*ϕ*, and 2) the 𝜅 used to generate ice crystal formation in the current simulation frame. For state *s* = [Δ*ϕ*, *κ*], we formulate porosity control by adjusting 𝜅 at evenly spread timesteps during the simulation. That is, given the initial state of the simulation *s*_0_ = [Δ*ϕ*_0_,*κ*_0_], the actor network uses *s*_0_ to output the action set *a* = [*κ*_1_,*κ*_2_,…, *κ*_*n*_], where *n* is the designated number of actions required. Using *a* and changing 𝜅 in accordance with the ice crystal growth prediction model, the state of the result of the prediction can be acquired as s_e_ = [Δ*ϕ*_e_,*κ*_e_]. We designed the reward function as the difference of the squared ice crystal rates, which can effectively penalize the agent if the ice crystallization rates become significantly different.

**Figure 7:**
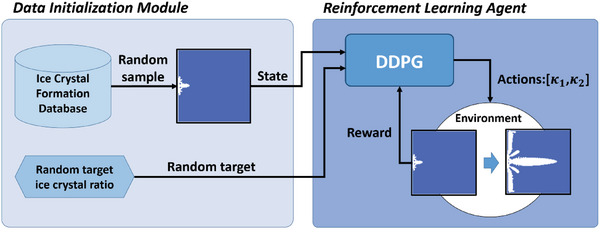
Training loop for proposed DDPG ice crystal control method. The data initialization module first samples random initial states from the ice crystal formation database and generates a random target ice crystallization rate. Then, the state and target are inputted into the DDPG algorithm using the random sample, the DDPG uses the actor network to acquire the corresponding actions using the initial state and target, and finally, the reward is calculated using the custom reward function to update the network. This process is repeated to train the DDPG.

To train the DDPG agent, we used the ice crystal formation database to initiate the prediction process as the environment of the algorithm, as shown in **Figure** **7**. A sub‐dataset of initial crystal formations was created by taking only the initial frames from the dataset. In each training episode, an initial crystal formation was sampled randomly from the sub‐dataset. Subsequently, a target ice crystallization rate of 0.3–0.95 is randomly generated and used as the ice crystallization rate that the environment should achieve. By converting the initial ice crystal formation into the designed state s_0_, s_0_ can be used as the input for the actor network to generate the action set *a* and also as the condition in the ResNet‐U‐net, resulting in the final state s_*e*_ and reward *r*. The episode tuple (*s*_0_,*a*, *r*, *s*_e_) is then saved in the replay buffer. By iterating this training process, porosity control of ice crystal growth can be achieved.

### 3.4 Two‐Stage Porosity Control Results

**Figure 8:**
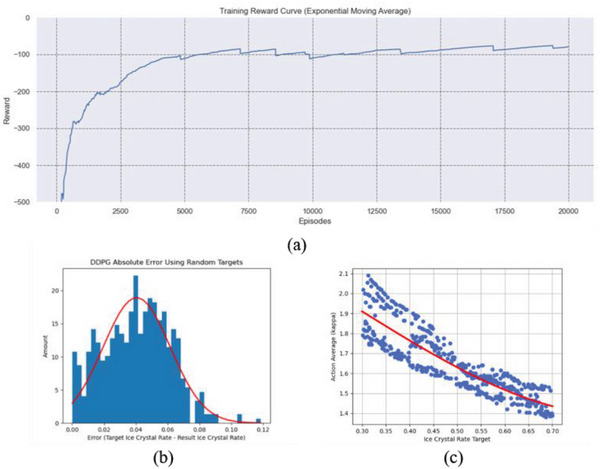
Results of the porosity control training. a) Resulting training reward curve of the proposed method; the agent converges at approximately 12 500 iterations. b) The error distribution over 500 randomly generated cases; the red curve is the approximated distribution of the resulting error, where the mean error is 0.118 and the standard deviation is 0.021. c) The action trend of the actor agent. Each data point represents an average of two 𝜅 values calculated using DDPG.

To fit the description of the ResNet‐U‐net, we tested our DDPG agent by adjusting 𝜅 twice during the simulation, forming a two‐stage porosity control model. The training parameters of the DDPG agent are summarized in **Table** **2** and the convergence of episodic reward can be referred to in **Figure** **8**a, where the open loop training converges after 20 000 iterations. After the model converged, we selected the model with the best average reward over 1000 episodes and used it as a testing model for the results.

**Table 2:** Parameters used to train the DDPG agent

Parameters	Value
Total training iterations	20 000
*γ*	0.99
*τ*	0.001
Replay buffer size	5000

To show the consistency and precision of the actor network of our trained DDPG agents, 500 cases of random initial states and ice crystal targets were tested, resulting in the error distribution shown in Figure 8b. The overall mean error between the target ice crystallization rate and the tested ice crystal rate is 0.041, and the standard deviation is 0.021. Additionally, even though the maximum error for this test is 0.118, there are less than five cases out of the total of 500, i.e., less than 1% of all cases, where the error is larger than 0.1. From these results we can infer that the trained DDPG agent has excellent accuracy and stability when controlling porosity, indicating the potential for wider applications of RL in the field of material design.

**Figure 9:**
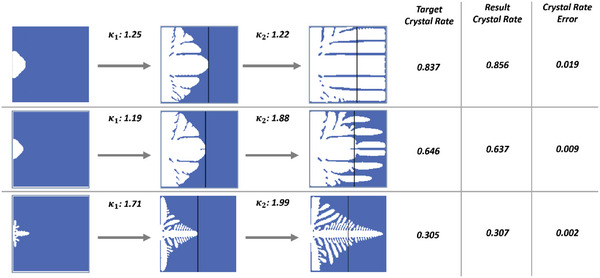
Results of the actor network and control of the DDPG agent. The simulation frames shown in the far‐left column are of the initial testing state. The column to its right is the result of the first controlled 𝜅_1_ the black line represents the border where the controlled parameter is 𝜅_2_ Finally, the simulation frames in the column to its right show the result of our control method. Observing the numerical results on the right‐hand side, the error between the target crystal rate and the result crystal rate is seen to be low.

Figure 8c shows the overall trend of the actions calculated by the actor, where each data point in the graph represents the averaged 𝜅 actions of each of the 500 cases. When describing the physical properties of 𝜅, we can know that it represents the dimensionless latent heat and is, therefore, proportional to it. Therefore, 𝜅 may affect the output of the simulation. When 𝜅 is larger, the dimensionless latent heat affects the overall simulation and reduces the crystallization rate. Figure 6c shows that, as the target increases, the average 𝜅 decreases. This phenomenon matches the physical properties of 𝜅. The results of our porosity control method can be observed in **Figure** **9**: through the three different target crystallization rates and initial crystal formations, our control method successfully controlled the porosity growth to within the target rates with high accuracy. It should be noted that when 𝜅 is low, the ice crystals formed during the growth process is denser than when 𝜅 is high. When the target crystal rate is higher, the calculated actions are lower, and vice versa.

## 4 Discussion

### 4.1 Deep Learning Approaches

In this study, dimensionless latent heat (𝜅) is used as a control condition to influence dendritic growth.^[^ ^25^ ^]^ We generate the training dataset by varying the 𝜅 value in the phase field modeling. By altering 𝜅, we can affect the crystallization speed and the resulting structure, i.e., the significant change in dendrites. During the simulation process, we adjust the 𝜅 value to modify the structure growth trend, and the deep learning model learns the association between varying 𝜅 and growth conditions. The trained model can then generate the next frame's result based on the current structure and the corresponding 𝜅 value. Consequently, we can use this model to create ice crystals with different parameter values. Dendritic ice crystals are also related to factors that impact freeze casting. In freeze casting, dendritic ice crystals form after cooling the material, and the ice crystals are subsequently removed to create porous materials. The properties of porous materials are influenced by the shape of the pores. As such, our design goal is to use porosity as a standard to control the RL algorithm, designing ice crystals according to the target we set.

Despite ice being one of the most common solid materials on Earth, our basic understanding of ice crystal growth remains strikingly insufficient.^[^ ^33^ ^]^ Earlier studies have attempted to improve phase field predictions using recurrent neural networks (RNN) such as long short‐term memory and the gated recurrent unit, predicting latent features for the phase field method.^[^ ^21^, ^34^, ^35^ ^]^ These precedents have leveraged the sequential prediction capability of RNNs. Using sequential structures produced by phase field simulations, these works used batches of initial structures, formed using the phase field method, to predict features in future time steps of the simulation. Some research has also used U‐net to predict specific timesteps of phase field simulation.^[^ ^22^ ^]^ However, what differentiates our model from these previous excellent works is our model's ability to exploit the existing parameter in the phase field method to control the outcome of the simulation. Furthermore, our work can predict the structure of future time steps with only a single image frame of the nucleus position and specific parameters as inputs. This work can potentially be applied in advanced materials manufacturing; such as freeze casting. With our ResNet‐U‐net, the large simulation time and computational cost associated with the conventional phase field method can be reduced to a great extent, which can be used as a surrogate model to accelerate the design framework.

Porosity plays an important role in the mechanical properties of porous materials. Previous attempts to control microstructures using RL^[^ ^35^ ^]^ and generative deep neural networks to optimize physical properties^[^ ^36^ ^]^ have been limited to specific formations and optimizations. Therefore, we propose using our ResNet‐U‐net as a surrogate for the phase field simulation to train an RL model to control porosity in simulation results. Since 𝜅 is included in our ResNet‐U‐net, this creates an environment that can be controlled by the intelligent agent in the RL algorithm. Using the DDPG method, we constructed an actor neural net that successfully controlled the overall growth conditions by adjusting the value of 𝜅. Although controlling porosity using 𝜅 as a parameter is common in real‐world applications, existing methods rely on empirical equations, operator experience, and costly simulations. We believe this method can offer insights into the possible applications of RL in material design, including reducing resource requirements.

There are various metrics that can be used to evaluate the quality of the ResNet‐U‐net and the RL model, but we believe our approach is appropriate for several reasons. Firstly, our model output is an image frame that distinguishes the liquid and solid parts of the structure, which can be seen as a binary classification task. Therefore, BCE loss is suitable for this task as it maximizes the likelihood between the individual pixels in the output data and the ground truth, providing excellent asymptotic properties for training. Moreover, BCE loss allows the model to train its accuracy both spatially and temporally since it determines the results frame by frame. This obviates the need for additional metrics like the growth rate, as the model trains itself to make better predictions in both spatial and temporal dimensions.

On the other hand, we used porosity as the metric to control the RL algorithm because it is practical in real‐world scenarios and has a direct impact on the mechanical properties of porous structures. Furthermore, it has a fast inference time as our image frame is binary and fixed in size. Porosity can be easily calculated for each frame without considering features across different frames. However, we are aware that other metrics can be used to optimize different features in future studies. Our success in controlling porosity using RL confirms the potential of our approach to design materials with specified properties, making it a promising method in the field of material design.

In our study, we used the phase field method to generate a dataset and trained our model using this dataset. The model demonstrated an excellent predictive ability for the growth of ice crystals and the control of crystal form, which can be useful in managing the production of various ice crystals. However, implementing this practically poses significant challenges due to the involvement of many complex variables in freezing, such as interactions with solutes and cooling methods. Each of these variables is significant enough to affect the results of freezing. Furthermore, while our study has demonstrated great predictability in the phase field results, further research and experiments are required to enable the model to output the temperature field. In phase field modeling, the phase and temperature fields are closely correlated with the generation of crystallization results. However, because of the nature of the phase field, the ResNet‐U‐net is optimized as a classification task. In contrast, the temperature field requires a precise prediction of temperature levels, making it a regression‐oriented task. If the temperature field is to be included as an additional channel in the output, the complexity of the model will increase since it will include both classification (phase field) and regression (temperature field) tasks in the output layer. As our primary goal was to investigate the design of the structure from phase field modeling, we focused our model on predicting the phase field. Nevertheless, with our successful result in the phase field, we believe that it is possible to achieve outputting both channels in future works.

In this study, we utilize the Kobayashi phase field model for the dataset to streamline and optimize the data generation pipeline. Given that this earlier proposed model forms the foundation for much of the subsequent work in the phase field modeling community, we consider it an ideal starting point for evaluating our method's performance. Although there are currently models that take into account the thin interface limit and numerous other improvements, our focus remains on the growth problem, which is inherently related to space and time.^[^ ^37^, ^38^, ^39^, ^40^, ^41^ ^]^ As the simulation process is influenced by the current parameters, both the current phase field and parameter changes become the primary factors affecting the outcome. Despite using only one image for prediction, the problem of predicting phase field modeling conditions also involves spatial and temporal spaces. Provided that the generated images exhibit a certain correlation in space and time steps, our ResNet‐U‐net model can predict the suitable structural morphology through parameter changes.

**Figure 10:**
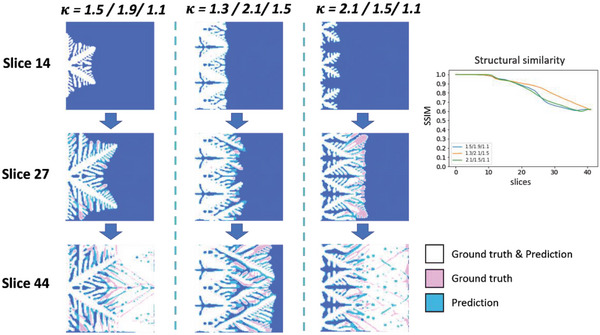
The deep learning model's prediction results, trained using the sixfold symmetry dataset, maintain the same parameters as the fourfold symmetry model. This model can also learn the relationship between 𝜅 and growth. However, due to the increased image complexity and the accumulation of iterative errors in the sixfold symmetry, its accuracy does not match that of the fourfold symmetry. Despite this, the overall characteristic trend remains consistent.

Owing to the strong reliance of deep learning on data, the results of current study are significantly influenced by the dataset used for training. In this case, we employed a sixfold symmetry dataset to test the model's capabilities, while keeping the parameters identical to those in the fourfold symmetry. This dataset consists of 2 variations in 𝜅, 44 frames, and a total of 636 cases. **Figure** **10** shows the prediction results for 3 cases, demonstrating that the model can also learn the relationship between the 𝜅 value and growth.

It is noticeable that the cumulative iterative errors in the sixfold symmetry do not align with those of the fourfold symmetry, primarily due to the increased image complexity. However, the overall trend remains consistent, and by modifying the conditions, the model can generate ice crystals with the corresponding features. This suggests that our deep learning model is capable of learning the relationship between input parameters and growth based on the provided dataset. The applicability and scalability of the model allow its application to datasets generated through a variety of methods.

### 4.2 Dendrite Structure Characteristics

Our study presents a preliminary framework in which dendrite growth is influenced by a single parameter 𝜅 during the growth process, utilizing deep learning. The growth of side branches is affected by noise or parameter changes in the simulation.^[^ ^42^, ^43^ ^]^ Traditionally, side branches develop by introducing noise in phase field simulations, which destabilizes the tip interface, resulting in diverse side branches. Although noise is frequently discussed in phase field model papers, we opted not to include it in this study. Our aim was to maintain control conditions focused on 𝜅 without being influenced by additional noise, leading to more robust and reliable results.

Side branches also occur due to state changes during the growth process, known as induced side branches. These branches are based on changes in interface temperature, anisotropy, and interface velocity in the Gibbs Thomson equation. The effect of anisotropy was investigated in a study by Gilles Demange et al.^[^ ^44^ ^]^ The magnitude of anisotropy influences side branch curves, causing them to be faceted or smoothed out. In cases where the change in anisotropy is discontinuous, initially, when the smoothness is minimal and the anisotropy shifts to a larger value, the change is not immediately visible. However, after a few time steps, the side branch curves become multifaceted. This demonstrates a process that induces side branches caused by long‐term perturbations of growth conditions. This process also aligns more closely with how we modify parameters in an iterative manner through deep learning models.

In our study, we observed that variation of parameters in the phase field model can lead to the formation of different side branches. We used a range of 𝜅 values, from small to large, resulting in structures ranging from plate to dendrite and then to a needle. As the value of 𝜅 increases, the main branches become thinner and more branched, and the fronts increase more slowly. In addition to 𝜅, other parameters also affect the structure's trend. For example, Kobayashi's work showed that anisotropic strength (*δ*) has an impact. When *δ* = 0, the top curve takes the shape of a semicircle, while *δ* = 0.02 forms a typical dendritic structure. *δ* = 0.05 leads to the development of vertical branching on the side, resulting in a significantly different structure. Another parameter that affects branch growth is the anisotropic gradient energy coefficient (*ε*). Yi Dang et al.^[^ ^40^ ^]^ found that a smaller *ε* leads to thicker main branches, whereas a larger *ε* leads to thinner main branches with a faster dendrite growth rate and denser overall structure growth. Different parameters in the phase field model have different effects and trends on structure growth. Our model can predict the effect of a single parameter. To make a deep learning model consider the trend of each parameter simultaneously, we need to use more diverse data distributions and modify the model design, enabling the model to learn and predict the structure that conforms to the trend through differences in parameter changes.

In our work, we influence the growth of the structures by adjusting the value of 𝜅 in the phase field model. 𝜅 is directly proportional to latent heat and inversely proportional to cooling intensity, and supercooling is positively related to crystallization temperature and melting point. In actual experiments, the degree of supercooling is affected by pressure. By altering 𝜅, we were able to influence the rate of crystallization, leading to significant changes in the structure. The datasets used for model training contain various 𝜅 value variations. The model operates by understanding the current phase field and the resulting change in the phase field corresponding to 𝜅, which includes the degree of crystallization and the shape of the side branches in the ice crystal structure. This aligns with our expectation that the model would approximate the phase field simulation process by predicting the next growth step of the phase field based on the current state. Although numerous conditions or parameters affect the final result of phase field modeling, our method employs ResNet‐U‐net and uses 𝜅 input as the control condition, demonstrating that deep learning has the potential to consider multiple parameters in the model to predict phase field variations.

There is still much work to be done on this topic. This study focuses on a 2D case but many papers related to the simulation of 3D dendritic growth have also been proposed.^[^ ^45^, ^46^, ^47^, ^48^ ^]^ To progress toward 3D predictions, the dimension of input images must be expanded to 3D, and additional features such as ice faceting, basal, and prismatic growth should be taken into account. Surface features containing radial ridges divide ice crystals into sectors, while concentric ribs appear to exhibit variations in crystal edge growth.^[^ ^49^ ^]^ Moreover, different column types also display variations in shape and length. More distinct forms, such as capped columns, feature large plates at their ends.^[^ ^37^ ^]^ A 3D representation can display the details of the ice structures in each dimension. However, to fully capture these characteristics, a higher resolution is necessary. Increased resolution brings about greater challenges in data generation, as well as in model design and training. Although the implementation of AI in dendritic growth remains a challenge, it is still seen as capable of providing some valuable insights. Moreover, controlling and designing porosity is desirable. With the experience that our team has accumulated in recent years in the field of RL,^[^ ^50^ ^]^ we remain positive about the ability of RL to address this problem.

## 5 Conclusion

A deep generative model was proposed to better understand the physics underlying ice morphology development and an RL algorithm was used to control ice crystal growth. The model is based on U‐net and ResNet and can solve the temporal‐spatial issues associated with the problem. Using a numerical method and the phase field method, we generated a variety of datasets related to the development of ice morphology using MATLAB, with adjustments made for the appropriate factors. We imported these datasets as an input sequence into our deep learning model. During the training process, the deep learning model extracts essential features of the input data and predicts the ice crystallization development trend in the subsequent processes. The development of ice crystallization at the next timestep can be predicted by the pretrained model during generation by analyzing the ice crystal morphology at the current time. The entire ice crystal growth process can be characterized by iterating the above operations. Furthermore, a DDPG algorithm is used to achieve control of the ice crystal growth using the developed ResNet‐U‐net as the environment. By formulating the state, actions, and reward functions, we successfully achieved porosity control with excellent accuracy and stability.

## Conflict of Interest

The authors declare no conflict of interest.

## Data Availability

The data that support the findings of this study are available from the corresponding author upon reasonable request.
